# Progression of Tubulointerstitial Fibrosis and the Chronic Kidney Disease Phenotype – Role of Risk Factors and Epigenetics

**DOI:** 10.3389/fphar.2017.00520

**Published:** 2017-08-08

**Authors:** Timothy D. Hewitson, Stephen G. Holt, Edward R. Smith

**Affiliations:** ^1^Department of Nephrology, The Royal Melbourne Hospital, Melbourne VIC, Australia; ^2^Department of Medicine, The Royal Melbourne Hospital, The University of Melbourne, Melbourne VIC, Australia

**Keywords:** kidney disease, fibrosis, fibrogenesis, epigenetics, autocrine, TGF-β1

## Abstract

Although the kidney has capacity to repair after mild injury, ongoing or severe damage results in scarring (fibrosis) and an associated progressive loss of kidney function. However, despite its universal significance, evidence highlights a population based heterogeneity in the trajectory of chronic kidney disease (CKD) in these patients. To explain the heterogeneity of the CKD phenotype requires an understanding of the relevant risk factors for fibrosis. These factors include both the extrinsic nature of injury, and intrinsic factors such as age, gender, genetics, and perpetual activation of fibroblasts through priming. In many cases an additional level of regulation is provided by epigenetic mechanisms which integrate the various pro-fibrotic and anti-fibrotic triggers in fibrogenesis. In this review we therefore examine the various molecular and structural changes of fibrosis, and how they are influenced by extrinsic and intrinsic factors. Our aim is to provide a unifying hypothesis to help explain the transition from acute to CKD.

## The Pathology of Acute Kidney Disease

When a mild or an acute kidney injury (AKI) occurs, tissue repair mechanisms are usually able to restore function. This wound repair consists of consecutive but overlapping events; inflammation, extracellular matrix (ECM) synthesis (fibrogenesis), resolution, regeneration, and remodeling (Hewitson, 2009). While there are subtle differences in pathogenesis, in general, injury results in an acute neutrophil and monocyte infiltration, which is over time replaced by macrophages. Re-epithelialisation is predicated upon resolution of inflammation, which includes amongst other signals a switch from a pro-inflammatory macrophage phenotype (M1) to a pro-repair one (M2). In each nephron segment the regenerative capacity depends on distinct epithelial lineages, and requires stabilizing scaffolds to guide reconstitution (Suarez-Alvarez et al., 2016). An important point therefore is that the underpinnings of fibrosis begin as a necessary and well-organized attempt to stabilize tissue through maintaining basement membranes and structural integrity for repair and regeneration.

## Exaggerated Fibrosis Accompanies Chronic Kidney Disease

If repair mechanisms are disrupted, or the injury-causing stimulus persists, AKI can progress into a chronic disorder, characterized by non-recoverable organ remodeling and scarring (fibrosis) (Hewitson, 2009). Histologically this process presents itself as glomerulosclerosis, vascular sclerosis and tubulointerstitial fibrosis, with the last being the best predictor of deteriorating renal function, regardless of etiology (Hewitson, 2009). This transition has led many to conclude that there is a ‘point of no return,’ a stage from which recovery and repair is no longer possible (Ito et al., 2004). Fibrosis exacerbates progression through capillary rarefaction and subsequent tissue hypoxia (Darby and Hewitson, 2016) while hypoxia itself in turn directly stimulates further fibrogenesis (Nangaku, 2006). Renal parenchymal fibrosis is therefore a failure of repair, and is a final common pathway in all progressive renal disease.

## Cellular Basis of Tubulointerstitial Fibrosis

In this review we focus on the pathogenesis of tubulointerstitial fibrosis due to its universal significance, and resemblance to fibrosis in general.

Experimental models and patient renal biopsy studies of tubulointerstitial fibrosis have repeatedly shown that activation of tubule epithelial cells and interstitial fibroblasts is responsible for excess ECM production, in particular collagen, which constitutes scar tissue. Injured epithelial cells synthesize collagen, which manifests itself as both basement membrane thickening (collagen IV) and interstitial fibrosis (collagens IV, I). Damaged tubules also release cytokines and pro-fibrotic signals that activate adjacent fibroblasts (Frank et al., 1993). The other key event in this process is an exponential increase in the number of fibroblasts after injury (Hewitson et al., 1995). These cells originate from not only resident fibroblasts, but also from renal tubules through epithelial mesenchymal transition, pericytes, circulating progenitors (reviewed in Hewitson, 2009), and even macrophages (Wang et al., 2017). The “fibroblasts” that accumulate during kidney disease are therefore a heterogeneous population of cells, which have been difficult to characterize cyto-chemically. Although a number of putative fibroblast markers have been identified, most investigators have associated *de novo* expression of alpha-smooth muscle actin (αSMA) with an activated phenotype. This so-called myofibroblast is characteristically hyperproliferative, contractile and fibrogenic. Similar phenotypic transitions occur in tubular epithelial cells (Darby and Hewitson, 2007) and glomerular mesangial cells (Johnson et al., 1991), highlighting the universal applicability of this process.

## Fibrogenesis is Cytokine Driven

At the molecular level, fibrogenenic cell activation is a predominantly cytokine driven process. Signals can be specific to the injury or derived from the uremic milieu systemically. Regardless, in each case the AP1 transcription factor c-Jun seems to be a central molecular mediator of fibroblast activity in multiple organs (Wernig et al., 2017).

**FIGURE 1:**
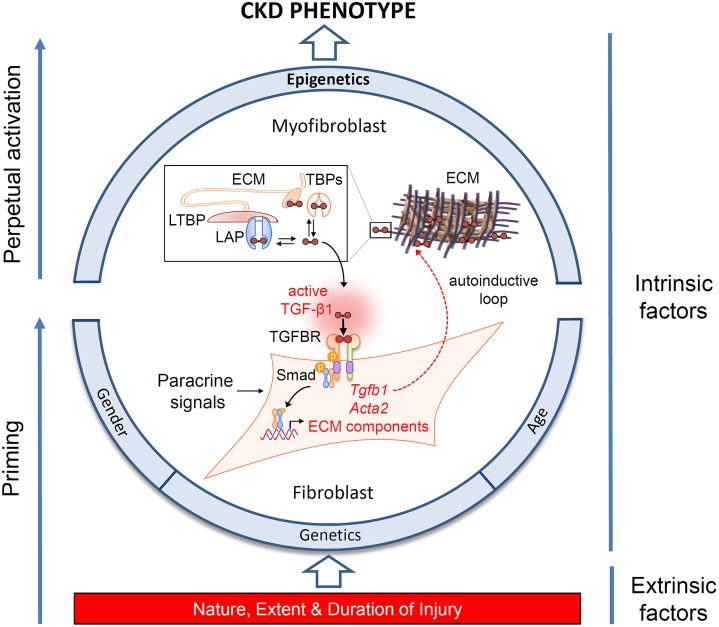
Determinants of the chronic kidney disease (CKD) phenotype. Schematic representation of the interplay of extrinsic and intrinsic risk factors in the progression of CKD, and their effects on myofibroblast differentiation. Fibroblast recruitment and activation is under control of paracrine and autocrine signals released in response to injury. The schema highlights the significance of an autocrine amplification of transforming growth factor beta1 (TGF-β1) signaling in injury-primed cells and their perpetual activation through epigenetic mechanisms. TGF-β1 is secreted as an inactive dimeric complex with latency associated peptide (LAP), bound in turn to latent TGF-β binding protein (LTBP). These complexes are subsequently incorporated into the extracellular matrix (ECM) and must be cleaved to release active TGF-β1. TGF-β binding proteins (TBPs) abundant in the extracellular fluid (e.g., decorin, betaglycan, fucoidan, heparin) also sequester TGF-β1, limiting activation at remote sites. Structurally, diffusion of TGF-β1 is also restricted by tubule basement membranes (not shown). Activation is therefore constrained to the fibroblast cell surface, where multi-step proteolysis releases TGF-β1 and allows binding to its cognate receptor complex (TGFBR) in an exclusively autocrine fashion. Canonical signaling via Smad phosphorylation drives the expression of intracellular α-smooth muscle actin expression (*Acta2*), ECM synthesis and further TGF-β1 gene expression generating an autoinductive feed-forward loop.

Since the landmark studies of Border and colleagues demonstrating a role for transforming growth factor beta 1 (TGF-β1) in glomerulosclerosis (Border and Ruoslahti, 1990), a multiplicity of evidence has implicated TGF-β1 as the pre-eminent fibrogenic cytokine. This has been supported by demonstrating both direct fibrogenic action and benefits from targeting TGF-β1 pathways pharmacologically (reviewed in Meng et al., 2016). Despite the established significance of TGF-β1 in fibrogenesis, controversies continue to exist. Translation to clinically useful therapies based on targeting this molecule have been uniformly disappointing (Voelker et al., 2017), thought to be due to the other pleotropic properties of TGF-β1 (Meng et al., 2016). Ablation of the TGF-β receptor ameliorates fibrosis in some studies (LeBleu et al., 2013), but not others (Neelisetty et al., 2015). While these discordant, and sometimes unexpected results, need to be viewed in context of the overall evidence, they have led us to reappraise the significance of TGF-β1 and how it acts. Foremost is the recognition that TGF-β1 is an autocrine factor acting on the resident and infiltrating inflammatory cells that produce it. TGF-β1 is secreted in a latent form in complex with latency associated peptide (LAP). LAP is itself disulfide linked to a further protein, latent TGF-beta binding protein (LTBP), which targets latent TGF-β1 to the matrix after secretion. A number of pathological features including proteases, oxidative stress, integrins (Annes et al., 2003) and changes in ionic strength (Lawrence et al., 1985) can release active TGF-β1 through cleavage and conformational changes in bonding. Paracrine actions are therefore severely limited by formation of the latent TGF-β1 complex immediately adjacent to each cell, and which cannot readily traverse the basement membrane (Venkatachalam and Weinberg, 2015) (**Figure 1**).

Additionally, other cytokines and growth factors, such as platelet derived growth factor (PDGF) (Johnson et al., 1998; Buhl et al., 2016) and angiotensin II (Ruster and Wolf, 2006), are also pro-fibrotic in a paracrine fashion (**Figure 1**). Recent findings also suggest that the paracrine fibroblast growth factor 23 (FGF23) signaling may be relevant to fibrogenesis. FGF23 was originally described as a bone-derived member of the endocrine FGF family known to regulate mineral handling. Circulating levels of this hormone rise early in chronic kidney disease (CKD) and are predictive of disease progression. Whilst the factors driving this increase from bone are partially understood, *de novo* expression in the kidney has also been noted following injury, leading to speculation about its functional significance in this context (Smith, 2014). Trying to isolate a fibrogenic role for FGF23 has been problematic due to the systemic changes in its synthesis and other mineral factors that occur in CKD. In this regard unilateral ureteric obstruction (UUO) has been particularly useful, as fibrogenesis in this model does not involve changes in bone-mineral parameters, and the presence of an intact contralateral kidney means that the animals are also not uremic. Using this model we have recently identified temporal and spatial increases in distal tubular FGF23 expression in early renal fibrosis, and demonstrated that FGF23 increases myofibroblast differentiation and fibrogenesis in a dose related fashion *in vitro* (Smith et al., 2017).

## Factors Affecting the Progression of Fibrosis

**FIGURE 2:**
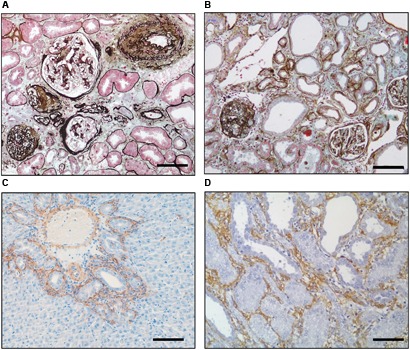
Inherent similarities between fibrosis in different species and organs. Silver–Masson trichrome staining of a diabetic **(A)** human and **(B)** rat kidney showing the underlying similarity of fibrosis in the two organs. Immunohistochemical staining for αSMA (brown) showing myofibroblast recruitment in the rat after**(C)** bile duct ligation in the liver and **(D)** unilateral ureteric ligation in the kidney. Scale bar = 100 μm. [Derived with modification from Hewitson (2012)].

The importance of several modifiable and unmodifiable risk factors (**Figure 1**) is borne out in epidemiological studies which show that the likelihood of progression in humans can be modeled by variables including age, gender and baseline renal function (Tangri et al., 2016). Fortunately our understanding of the cellular and molecular role of these factors has been aided by the fact that the final endpoint of fibrosis is remarkably similar in different species and etiologies (**Figure 2**), meaning that we have robust and reproducible experimental models to investigate specific risks for progression of fibrosis.

### Nature of Injury

The causes of kidney disease are diverse and include immunological, genetic, infectious, metabolic, physical and hemodynamic stresses amongst others. In each case the degree of fibrosis is determined in part by the extent of the damage, and the duration of injury, with severity and frequency of tubule injury being shown to determine prognosis (Takaori et al., 2016). Consistent with this, severity of AKI predicts progression to CKD (Ishani et al., 2009). The loss of basement membrane in severe injury is a major impediment to repair as it represents a loss of a scaffold for regeneration. The failure of injured epithelium to regenerate and repair results in glomerulosclerosis and tubular atrophy (Hewitson, 2009). We also know that the loss of regenerative capacity in tubular epithelial cells after injury corresponds to arrest in the G2/M phase of the cell cycle (Yang et al., 2010). Prolonged G2/M arrest triggers a pro-fibrotic phenotype in cultured tubule epithelial cells with a corresponding increase in TGF-β1, connective tissue growth factor (CTGF) and collagen IV mRNA transcripts (Yang et al., 2010).

### Perpetual Activation of Fibroblasts

It has long been recognized that fibroblasts cultured from human kidneys with interstitial fibrosis grow at a faster rate and produce more collagen than those derived from normal kidneys, and that these changes are passed on from passage to passage (Rodemann and Muller, 1990). A key feature in parenchymal scarring therefore lies in the perpetual activation of myofibroblasts. This has parallels in the skin where extracellular signal-regulated kinase (ERK) is found to be constitutively activated in dermal fibroblasts isolated from patients with scleroderma (Asano et al., 2005). In this case an upregulated expression of α_v_β3 integrin in fibroblasts from scleroderma compared to normal dermal fibroblasts provides an autocrine loop through its actions as a receptor for adjacent latent TGF-β1 (Asano et al., 2005). Our observations in rat renal fibroblasts are also consistent with a priming of cells during injury, making them more susceptible to fibrogenic signals, a difference that is maintained across generations in culture. In our recent study, we found that while FGF23 enhances TGF-β1 signaling in fibroblasts from kidneys with UUO, it failed to activate fibrogenic pathways in those derived from normal kidneys (Smith et al., 2017). Further analysis revealed greater FGF and TGF receptor density on fibrotic fibroblasts compared to their normal counterparts, and a feed forward induction of TGF-β1 expression and activity by FGF23 (Smith et al., 2017).

### Age

A progressive decline in renal function is common with aging (Weir, 1997), albeit with wide variability. Both hemodynamic and structural changes occur (Zhou et al., 2008) and aging rats can be shown to have impaired redox homeostasis (Aydin et al., 2012) and angiogenesis (Kang et al., 2001). Replicative senescence through telomere shortening is seen (Zhou et al., 2008), although the phenotype of telomere deficient mice confirm that it is not the only relevant factor (Schildhorn et al., 2015). Taken together these changes predispose older kidneys to new acute organ injury (Liu et al., 2017), as well as exacerbating progression of CKD. Likewise, there is evidence to suggest that the kidneys of elderly people are more sensitive to primary and secondary renal disease (Razzaque, 2007; Ishani et al., 2009).

### Genetics

Approximately 25% of incident dialysis patients have close relatives with CKD (Freedman et al., 2005), and the distinct susceptibilities of different rodent strains to experimental CKD strongly suggests that genetic variations impact renal fibrogenesis (Kokeny et al., 2009). Likewise, familial clustering and disparities in prevalence of CKD across race suggest a strong genetic component to progression (Uwaezuoke et al., 2016). However, identifying relevant polymorphisms in human kidney disease has been somewhat disappointing to date (Tampe and Zeisberg, 2014). Even though there are strong associations between single nucleotide polymorphisms and incident CKD, the association with end stage kidney disease, and therefore progression, is poor (Boger et al., 2011).

### Gender

Many studies have also shown a gender basis to progression of senescence and CKD, with epidemiological studies showing that females have a lower prevalence and slower rate of progression than males (Yu et al., 2010). Consistent with this, bioactive estrogen metabolites both prevent renal collagen synthesis *in vitro* (Lei et al., 1998) and reduce glomerulosclerosis and interstitial fibrosis *in vivo* (Maric et al., 2004). Nevertheless other investigations suggest that the male predominance is due to detrimental effects of testosterone (Baylis, 1994; Hewitson et al., 2016), rather than the protective effects of estrogen.

## Epigenetic Regulation of Fibrosis and Progression

While risk estimates based on the above factors allow accurate discrimination of those who are likely to progress over a 5-year period (Tangri et al., 2016), they do not explain the marked heterogeneity in the trajectory to renal failure frequently encountered (Li et al., 2012; O’Hare et al., 2012). Moreover, it is uncertain whether these factors capture risk over longer time horizons and developments in therapeutic options targeting these factors have been limited. This has led to the search for additional pathways that might modulate a person’s risk. A major shift in our understanding of fibrosis has been the recognition that epigenetic mechanisms operate at an additional level to integrate the various intrinsic and extrinsic pro-fibrotic triggers and fibrogenesis (**Figure 1**).

Epigenetics refers to stable changes in gene activity that are heritable in cell division, but do not involve changes in the DNA sequence (Reddy and Natarajan, 2015). Epigenetic modifications and consequential changes in protein expression have been described in diverse forms of renal disease including renal cancer, transplantation, autoimmune disease, and diabetes (reviewed in Chmielewski et al., 2011). Under normal conditions epigenetic modifications are balanced and reversible, but this may be disrupted in disease.

Often referred to as a second genetic code, epigenetic regulation of renal gene expression involves multiple mechanisms including post-replicative DNA methylation (Bechtel et al., 2010), RNA interference (McClelland et al., 2015; Gomez et al., 2016) and post-translational histone modifications (Wing et al., 2013).

Epigenetic regulation can occur via direct modification of genomic DNA. In this case DNA is methylated by the attachment of a methyl group to the 5′ position of cytosine residues in specific regions of DNA where cytosine and guanosine are separated by a single phosphate group (CpG sites). CpG methylation generally leads to transcriptional repression of genes (Mann and Mann, 2013). Interestingly, Bechtel et al. (2010) have identified several DNA methylations unique to fibroblasts derived from fibrotic kidneys. Epigenetic silencing of RASL1, a suppressor of the proto-oncogene Ras, results in persistent activation of fibroblasts. Importantly their time course studies also suggested a physiological reversible activation with short-term exposure to TGF-β1, and an irreversible methylation of the RASL1 promoter with long-term TGF-β1 exposure, providing a mechanistic rationale for priming.

Epigenetic silencing also occurs via non-coding micro RNAs (miR) which are small (19–25 nucleotides) RNA molecules that cleave or translationally repress targeted mRNA, and are well-described in kidney disease (Gomez et al., 2016) and transplantation (Jelencsics and Oberbauer, 2015). Various miR have a specific role in regulating ECM production, with both anti-fibrotic and pro-fibrotic actions through aberrant TGF-β1 signaling (Meng et al., 2016). Specific examples include pro-fibrotic miR-21 (Denby et al., 2011; Kolling et al., 2017) and miRNA-130 (Bai et al., 2016), and anti-fibrotic miR-29 (Liu et al., 2010). Actions may also be pleiotropic and context specific (Jelencsics and Oberbauer, 2015) with miR-192 being both anti-fibrotic and pro-fibrotic depending on local circumstances (Chung et al., 2010). miR-132 is of particular interest as it has recently been shown to counteract progression of renal fibrosis *in vivo* by selectively inhibiting myofibroblast proliferation (Bijkerk et al., 2016).

Recent attention has focused on the significance of post-translational histone modifications (marks) in the kidney. Nucleosomes consist of chromosomal DNA wrapped around core histone subunits (H2A, H2B, H3 and H4). These histones also have a flexible N-terminus tail of amino acids that extends from the nucleosome and can be post-translationally modified by phosphorylation, sumoylation, ubiquitination, acetylation (Ac), and methylation (Me). Modifications to the amino acid Lysine (K) on H3 and H4 histone tails have proven to be particularly relevant in fibrogenesis. Acetyl groups are added by the actions of various acetyl transferases (HAT) and removed by histone deacetylases (HDAC), with net enrichment a balance between the activities of these enzymes. Likewise, methylation is under the control of various methyl transferases (MHT) and their corresponding demethylases (HDM). Unlike acetylation however, more than one methyl group can be added meaning that histones can be mono- di- or tri- methylated. These histone modifications are docking sites for various proteins including co-activators, co-repressors, chromatin remodeling proteins. Their interaction with specific gene promoters form a pattern that regulates transcription (Allis and Jenuwein, 2016). Enrichment of H3KAc is often, but not exclusively, associated with relaxed chromatin and active gene expression, while H3K methylation can either activate or repress transcription depending on the Lysine involved, and the degree of methylation (Allis and Jenuwein, 2016).

Global histone methylation and acetylation are consistent features in fibrosis. TGF-β1 induces time dependent increases in methylation of K9 on H3 in renal fibroblasts *in vivo* (Sun et al., 2010). Histone marks regulate fibroblast differentiation in a variety of fibrotic pathologies (Guo et al., 2009; Mann et al., 2010; Zhou et al., 2010; Cho et al., 2012; Perugorria et al., 2012) including the kidney (Irifuku et al., 2016). More specifically, TGF-β1 exposure can both enrich active histone marks, and decrease repressive marks, at various pro-fibrotic gene promoters in renal mesangial cells (Sun et al., 2010; Yuan et al., 2013), epithelial cells (Sasaki et al., 2016), and fibroblasts (Irifuku et al., 2016; Sasaki et al., 2016). Histone phosphorylation at H3Ser10 seems to contribute to the G2/M arrest in tubule epithelia, although its target gene remains unknown (Yang et al., 2010).

Despite their potential significance, defining the role of histone marks will not be easy for a number of reasons. Rather than a code *per se*, it is probably the combined pattern of various histone marks that is relevant. Furthermore, our own studies show that even when there is no overall quantitative change in the prevalence of a mark, there are nuclear re-distributions of individual marks in response to TGF-β1, indicative of the dynamic state of histone modifications (Hewitson et al., 2017). We therefore need to better delineate the interaction of the various acetylation/methylation enzymes and their corresponding deacetylation/demethylation counterparts to better understand the basis of metabolic memory in fibrosis.

## Conclusion

While the kidney can recover from acute and limited damage, critical illness or a persistent injury leads to a complex chain of direct and indirect sequelae resulting in chronic progressive renal impairment without repair. Severe and ongoing injury, influenced by susceptibility and epigenetics, leads to priming, feed-forward induction, and perpetual activation of fibroblasts, and a failure to return to tissue homeostasis through repair. This model provides a unifying hypothesis helping explain the transition from AKI to CKD (**Figure 1**). A better understanding of specific modifiable risk factors will excitingly provide new targets for therapeutic intervention.

## Author Contributions

TH, SH, and ES contributed to writing of manuscript, gave final approval and are responsible for the work.

## Conflict of Interest Statement

The authors declare that the research was conducted in the absence of any commercial or financial relationships that could be construed as a potential conflict of interest. The reviewer BHA and handling Editor declared their shared affiliation, and the handling Editor states that the process met the standards of a fair and objective review.
